# Performance, Carcass Traits, and Meat Fatty Acid Profile of Post-Weaning and Finishing Zebu Steers on Tropical Pasture with Three Low-Intake Supplementation Strategies

**DOI:** 10.3390/ani14172486

**Published:** 2024-08-27

**Authors:** Diana Carolina Cediel-Devia, Luís Henrique Schaitz, Fabiano Ferreira da Silva, Laize Vieira Santos, Ana Paula Gomes da Silva, Marceliana da Conceição Santos, Wbeimar Yamit Sanchez Dueñez, Osman Ronaldo Aguilar Melgar, Tarcísio Ribeiro Paixão, João Wilian Dias Silva, Thiago Luís Alves Campos de Araújo, Dorgival Morais de Lima Júnior, Robério Rodrigues Silva

**Affiliations:** 1 Department of Rural and Animal Technology, Southwest Bahia State University, Primavera Square, Itapetinga 45700-000, BA, Brazil; diana.devia@estudante.ufla.br (D.C.C.-D.); luis_schaitz@hotmail.com (L.H.S.); ffsilvauesb@hotmail.com (F.F.d.S.); santos.laize@yahoo.com.br (L.V.S.); anazte@hotmail.com (A.P.G.d.S.); marciazootec@hotmail.com (M.d.C.S.); wbeimar.duenez@ufv.br (W.Y.S.D.); 94oram@gmail.com (O.R.A.M.); tarcisioirun@hotmail.com (T.R.P.); joaowiliand@yahoo.com (J.W.D.S.); rrsilva.uesb@hotmail.com (R.R.S.); 2 Department of Animal Science, Federal University of the Semi-Arid Region, Francisco Mota Street, Costa e Silva, Mossoró 59625-900, RN, Brazil; thiagotor4@hotmail.com

**Keywords:** protein–energy, protein supplement, supplementation level, urea

## Abstract

**Simple Summary:**

Protein and energy supplementation increases the performance of pasture-finished cattle. Three low-intake supplementation strategies (restricted, low, and moderate) were tested during the post-weaning and finishing steers on pasture for 310 days. The moderate supplementation strategy (use of 1 g/kg and 2 g/kg of body weight of concentrated supplementation during the rainy and dry seasons, respectively) increased the weight and fat content of the steers’ carcass and the intramuscular fat of the beef. However, steers consuming the moderate supplementation strategy produced meat with lower levels of omega-3 fatty acids. We recommend the use of 1 g/kg and 2 g/kg of body weight of concentrated supplementation during the rainy and dry seasons, respectively, in the post-weaning and finishing steers on tropical pastures.

**Abstract:**

The study aimed to evaluate the effects of three supplementation strategies on intake, apparent digestibility, feeding behavior, performance, carcass traits, proximate composition, and the fatty acid profile of meat from steers on tropical pasture during the post-weaning and finishing stages. The experiment involved 33 1/2 Holstein × 1/2 castrated Zebu steers weighing 335 ± 42.90 kg, aged 22 ± 2 m. The animals were managed on *Urochloa brizantha* cv. Marandu using an intermittent grazing system with continuous stocking and variable stocking rates for 310 days. The supplementation strategies were as follows: MS/US (mineral salt/urea supplementation): mineral salt in the rainy season and mineral salt with urea in the dry season; US/PS1 (urea supplementation/protein supplementation): mineral salt with urea in the rainy season and protein supplement at 1 g/kg body weight (BW) in the dry season; and PS1/PS2 (protein supplementation 1/protein supplementation 2): protein supplement at 1 g/kg BW in the rainy season and 2 g/kg BW in the dry season. The dry matter intake did not differ significantly (*p* > 0.05) between strategies. However, the post-weaning PS1/PS2 strategy resulted in higher (*p* < 0.05) crude protein intake. The final body weight did not differ (*p* > 0.05) between the strategies, but the average daily gain in post-weaning and finishing was higher (*p* < 0.05) for MS/US (restricted) animals. Carcass weight, subcutaneous fat thickness, and lipid content in meat were significantly higher (*p* < 0.05) for steers in the PS1/PS2 group. Steers finished on MS/US produced meat with a higher content of polyunsaturated fatty acids and ω-3 fatty acids (*p* < 0.05). Concentrate supplementation at 1 g/kg BW during the rainy season and 2 g/kg BW during the dry season is recommended for post-weaning and finishing steers on tropical pasture.

## 1. Introduction

Beef cattle production under grazing conditions is significantly influenced by seasonal variations in light, rainfall, and temperature [1]. These factors directly impact the chemical composition and availability of forage in the pasture, affecting the performance of grazing cattle [2]. In tropical regions near the equator, seasonal variations are more pronounced, typically divided into rainy and dry seasons [3]. During the dry season, pasture quality and availability decline, leading to a significant loss in nutritional value [4]. This is particularly true for protein, which is essential for fiber fermentation in the rumen of forage-fed animals [5].

Supplementing cattle on pasture is a well-established feed security strategy that aims to maximize forage utilization throughout the year, thereby enhancing the performance of post-weaning and finishing animals on pasture. Given the seasonal variations in forage chemical composition, post-weaning and finishing supplementation strategies must be adjusted accordingly [6]. This differentiation particularly applies to the type of nitrogen used (e.g., non-protein nitrogen, true protein, and rumen-undegradable protein) and the energy source (e.g., non-fibrous carbohydrates, fibrous carbohydrates, and sugars).

Rocha et al. [7] investigated two supplementation strategies during the post-weaning period for steers on tropical pasture: mineral supplementation during the rainy season with the protein concentrate at 1 g/kg body weight (BW) during the dry season, and the protein concentrate at 1 g/kg BW during the rainy season with 2 g/kg BW during the dry season. They found similar results in animal performance and feed conversion at the end of the post-weaning phase. Likewise, Ramos et al. [8] found that supplementing steers with protein concentrate during at least one season (either dry or rainy) produced a carcass and meat quality similar to the quality of those supplemented during both seasons.

Furthermore, there is a consensus that protein or protein–energy supplementation for steers during the post-weaning phase has a positive residual effect on performance and carcass and meat characteristics during the finishing phase. [9,10,11,12] reported higher ω-3 deposition and a lower ω-6:ω-3 ratio in the meat of steers finished with a mineral mixture only, compared to those supplemented with protein concentrate.

However, studies comparing strategies that use protein supplementation from non-protein nitrogen sources (such as mineral salt with urea) versus lower levels of protein–energy supplementation for post-weaning and finishing cattle on pasture are limited. Their impact on performance, carcass characteristics, and meat fatty acid profile remains an area for further research.

Our hypothesis was that a moderate adjustment in the supplementation of steers, with a nutritional strategy of 1 g/kg BW during the rainy season and 2 g/kg BW during the dry season, during the post-weaning and finishing phases, could lead to improved performance, carcass traits, and meat quality compared to a low-supplementation approach (mineral salt with urea during the rainy season and protein concentrate at 1 g/kg BW during the dry season) and a restricted approach (mineral salt during the rainy season and mineral salt with urea during the dry season).

Therefore, this study aimed to evaluate the impact of three supplementation strategies on intake, apparent digestibility, feeding behavior, performance, carcass traits, proximate composition, and fatty acid composition in meat from post-weaning and finishing steers on tropical pasture.

## 2. Materials and Methods

### 2.1. Animals, Treatments, and Design

The experiment was conducted in Ribeirão do Largo, Bahia, located at co-ordinates 15°26′46″ S and 40°44′24″ W, at an altitude of 800 m, with a Koppen Aw climate classification. The study duration was 310 days.

A total of 33 crossbred steers (1/2 Holstein × 1/2 Zebu) with an initial average weight of 335 ± 42.90 kg and an average age of 22 ± 2 months were used. At the beginning of the study, the animals were dewormed with Ivermectin (IVOMEC^®^, Merial, Cuiabá, Brazil) and immunologically castrated (BOPRIVA^®^, Pfizer, New York, NY, USA). The steers were divided into three groups of 11, with each group receiving a different supplementation strategy in a completely randomized design (Table 1).

The supplementation strategies were as follows: Restricted: mineral salt during the rainy season and mineral salt with urea during the dry season; Low: mineral salt with urea during the rainy season and protein concentrate at 1 g/kg BW during the dry season; and Moderate: protein concentrate at 1 g/kg BW during the rainy season and 2 g/kg BW during the dry season. The concentrate supplement was formulated according to the National Research Council [13] guidelines, with a target average daily gain of 0.6 kg/day (Table 2 and Table 3). It was provided daily at 10h00 in uncovered plastic troughs accessible from both sides, with a linear sizing of 80 cm per animal. Drinking water was available ad libitum from 500 L automatic drinkers in all paddocks.

### 2.2. Grazing Method and Forage Characteristics

The animals were managed in an intermittent grazing system with continuous stocking and a variable stocking rate. The total grazing area covered 11.85 ha of *Urochloa brizantha* cv. Marandu. To minimize possible discrepancies between paddocks that could impact animal performance, the experimental area was divided into three modules. Modules A and B comprised 14 paddocks, each half a hectare, while module C had five paddocks of approximately one hectare each. The experimental design was managed to provide a 35-day rest period for each paddock. To achieve this, the animals stayed in each paddock for five days in modules A and B, and for seven days in module C, resulting in a 35-day evaluation cycle. At the end of each cycle, the animals were rotated between modules to ensure consistent experimental conditions across all groups.

Forage samples were collected at 35-day intervals from the paddocks where animals entered and exited in each module. Forage availability was evaluated using the comparative visual yield method described by Haydock and Shaw [14]. The weight of the fresh forage mass from the square was obtained using a semi-analytical digital scale. Following this, the samples were separated into morphological components (dead material, stem + sheath, and leaf), each of which was weighed to determine its proportion.

Forage intake was estimated using a simulated grazing procedure as described by Johnson [15]. The potentially digestible dry matter content (pdDM) of the forage was calculated using the equation by Detmann et al. [16]:

pdDM = 0.98 [(100 − NDFap) + (NDFap − iNDF)](1)

where NDFap = neutral detergent fiber corrected for ash and protein; and iNDF = indigestible neutral detergent fiber. All components in the equation are expressed in percentage terms.

Forage allowance (FA) was calculated using the following equation:

FA = {[((FM entry + FM exit)/2)/SR]/N of days} × 100(2)

where FA = forage allowance (kg BW/ha); FM = forage mass (kg DM/100 kg BW); and SR = average animal load per period (kg BW/ha).

### 2.3. Intake and Apparent Digestibility

Intake and digestibility assessments for dry matter and nutrients were conducted between the 50th and 61st days of the experiment. Fecal output by the animals was estimated using the external marker chromic oxide (Cr_2_O_3_) at a dose of 10 g/animal/ day. This marker was administered daily at 06h00 in a single dose for 12 days, delivered in paper cartridges and inserted manually into the oral cavity of each animal. The first seven days were used to stabilize the marker’s excretion flow and to accustom the animals to handling, while the final five days were dedicated to feces collection. Feces were collected at alternating times (08h00, 10h00, 12h00, 14h00, and 16h00) to obtain samples representing the daily gastrointestinal flow.

Feces were collected daily, directly from the rectal ampulla of the animals, labeled, and stored in a freezer at −20 °C. To create a composite sample, 100 g of pre-dried feces were weighed per animal each day, then homogenized for further analysis. Fecal dry matter output was estimated using the INCT-CA M-005/1 method described by Detmann et al. [17]. To calculate fecal output with chromic oxide, the following equation was applied:

FO = (Cr_2_O_3_ supplied, g/day/Cr_2_O_3_ in feces, %) × 100 (3)

where FO = fecal output (g/day).

To estimate individual dry matter intake from the supplement, the external marker titanium dioxide (TiO_2_) was used at a dose of 15 g/animal/day. The marker was added and thoroughly mixed into the supplement, which was provided daily at 10h00 for 11 days, following the methodology by Titgemeyer et al. [18]. The concentration of titanium in the feces and in the supplement was analyzed using the INCT-CA M-007/1 method described by Detmann et al. [17]. The supplement dry matter intake was calculated using the following equation:

SDMI = ((FE × TiO_2_ in feces, g/day)/TiO_2_ in supplement, %) (4)

where SDMI = supplement dry matter intake (kg/day); and FE = fecal output (g/day).

The voluntary roughage intake was estimated using the internal marker iNDF (indigestible neutral detergent fiber), following the methodology of Detmann et al. [17]. This method involves the ruminal incubation of a 0.5 g air-dried sample of feces, concentrate, and forage, ground to a 2 mm particle size. Samples are taken in duplicate and placed in non-woven fabric bags (‘TNT’) with a grammage of 100 g/m² and dimensions of 5 × 5 cm. The ruminal incubation period lasted 288 h. The residual material was subjected to neutral detergent extraction to determine the iNDF content.

The forage dry matter intake was calculated using the following formula:

FDMI = [(FO × %iNDF in feces) − %Marker in the forage]/%Marker in supplement(5)

where FDMI = forage dry matter intake (kg/day); and FO = fecal output (kg DM/day).

The apparent digestibility coefficients (ADC) of nutrients were estimated using the following equation:

ADC, g/kg = [(NI − NE)/NI](6)

where NI = nutrient intake (g/day); and NE = nutrient excretion (g/day).

### 2.4. Chemical Composition of Forage, Supplements, and Feces

Samples of forage, concentrate, and feces were pre-dried in a forced-air oven at 55 °C for 72 h, then ground in a Wiley-type mill with a 1 mm sieve. The following analyses were conducted according Detmann et al. [17]: dry matter (DM; INCT method CA G-001/1); mineral matter (MM; INCT method CA M-001/1); crude protein (CP; INCT method CA N-001/1); ether extract (EE; INCT method CA G-004/1); neutral detergent fiber (NDF; INCT method CA F-002/1), with corrections for protein and ash (NDFap; INCT CA N-004/1 and CA M-002/1); acid detergent fiber (ADF; INCT method CA F-004/1), with corrections for protein and ash (ADFap; INCT CA N-005/1 and CA M-003/1); lignin (INCT method CA F-005/1); and indigestible neutral detergent fiber (iNDF, INCT method CA F-009/1). For forage and feces samples, the non-fibrous carbohydrates corrected for ash and protein (NFCap) were determined using the equation proposed by Detmann et al. [17]. The total digestible nutrient (TDN) content in forage and supplements was calculated using the formula from [19].

### 2.5. Feeding Behavior

Feeding behavior was evaluated by trained observers from the 85th to the 87th day of the experimental period, resulting in 72 continuous hours of data collection. The parameters collected included grazing time, rumination time, time spent at the trough, time spent on other activities, total feeding time, and total chewing time. Activities surrounding rumination, grazing, idling, and feeding at the trough were recorded at 5 min intervals in an ethogram to determine the time expended on each of these activities. Time-series discretization was performed directly in the data collection worksheets, by counting the discrete bouts of feeding, other activities, and rumination (Silva et al. [20]).

Rumination chews were visually assessed by observing rumination activities and counting the number of chews per cud. Observations were made three times in the morning (before the concentrate was provided) and three times in the afternoon for each animal, according to the procedure by Bürger et al. [21]. Additionally, the number of bites and the time (in seconds) spent grazing were measured, with three replicates per animal in the morning and three in the afternoon, according to the methodology described by Baggio et al. [22].

After determining the values for dry matter intake (DMI) and neutral detergent fiber (NDF) intake, the efficiencies for rumination and feeding were calculated using the formulae recommended by Silva et al. [20].

### 2.6. Performance and Carcass Traits

The average daily gain (ADG) during the experimental period was calculated by weighing the animals at the start and end of the experiment, following a 12 h water/feed fast for both measurements. The ADG was determined by dividing the total weight gained during the period by the number of days elapsed. Intermediate weighing sessions were performed every 35 days to adjust the amount of concentrate supplied, as it is based on the body weight of the animals. Feed conversion was calculated using the values for DM intake and ADG.

The animals were slaughtered at an average age of 33 months, with an average final live weight of 490.4 ± 50.8 kg. Before transportation to the slaughterhouse, they were fasted for 16 h, and then weighed to determine slaughter weight (SW). At this point, ribeye area and backfat thickness were measured using ultrasound with an ultrasound device (CTS-900V, Ultramedic, Boa Vista, Brazil).

After weighing and measurements, the animals were transported to a slaughterhouse-packing plant, where they were slaughtered according to the standard procedure and the flow of the plant, adhering to the guidelines of Normative Instruction no. 3, of 17 January 2000, from the Ministry of Agriculture, Livestock, and Supply. The carcasses were then halved and weighed to determine the hot carcass weight (HCW), labeled, and placed in a cooling chamber for 24 h at around 0 ± 0.5 °C. Hot carcass yield (HCY) was calculated using the following formula:

HCY = (HCW/SW) × 100(7)

### 2.7. Proximate Composition and Cholesterol Content of Meat

Meat samples, each weighing 0.5 kg, were extracted from the Longissimus thoracis (LT) muscle on the right side of the animals’ cold carcass, specifically between the 12th and 13th ribs. These samples were then divided into two pieces of about 0.25 kg each. They were wrapped in polyethylene film, then in aluminum foil, and finally placed in labeled plastic bags. The labels contained information about the animal, treatment, and type of analysis. The samples were immediately stored at −10 °C until laboratory analyses. After two months, the samples were thawed to measure moisture, ash, and protein content. The ash and protein analyses using method no. 923.03 and no. 920.87, respectively [23]. Total lipid extraction was conducted using a mixture of chloroform, methanol, and water in a ratio of 2:2:1.8 (*v*/*v*/*v*), following the Bligh and Dyer [24] method.

The cholesterol analysis in meat was performed following the procedure described by Saldanha et al. [25]. Cholesterol determination utilized a high-performance liquid chromatography (HPLC) system (Shimadzu) equipped with a degasser (DGU-20 A5), and two pumps (LC-20 AT, with a UV detector). A C18 analytical column (250 mm × 4.6 mm × 3.5 μm) was used for the separation. Cholesterol identification was based on the comparison of retention times between the samples and the standard, while quantification relied on the corresponding peak areas. The chromatographic data were processed with LabSolutions^®^ software 6.81 version (Shimadzu, Kyoto, Japan).

### 2.8. Fatty Acid Composition

The fatty acid composition analysis followed the methodology described by Bannon et al. [26]. Fatty acid methyl esters were analyzed using a Shimadzu GC-2010 Plus gas chromatography system (Shimadzu, Kyoto, Japan), equipped with a Flame Ionization Detector (FID) and an Rt-2560 fused silica capillary column (100 m, 0.25 mm i.d.). Gas flow rates (White Martins) were 40 mL/min for the carrier gas (H2), 30 mL/min for the auxiliary gas (N2), and 400 mL/min for the synthetic flame air. The sample split ratio was 90:10. The operating parameters were optimized to ensure the best resolution. The injector and detector temperatures were set at 225 °C and 260 °C, respectively. The column temperature program started at 140 °C for 5 min, followed by a ramp of 3 °C/min until 245 °C, where it was held for 20 min, resulting in a total analysis time of 60 min. Injections were performed in duplicate with a volume of 1.0 μL each. Peak areas of fatty acid methyl esters were determined using GCSolution^®^ software 6.81 version.

Fatty acid methyl esters were identified by comparing the retention times of sample constituents with those of a mixture of fatty acid methyl ester standards (Mix C4-C24-18919-1 AMP, Supelco, MilliporeSigma, Burlington, MA, USA). Additionally, comparisons were made with standards containing the c9-t11 and t10-c12 geometric isomers of linoleic acid (O-5632 Sigma, MilliporeSigma, Burlington, MA, USA).

Fatty acids from fresh meat were quantified in g/100 g of total lipids using the internal standard methyl tricosanoate (23:0) (Sigma, USA). After weighing the lipids (~150 mg) for transesterification, 1000 μL of the internal standard solution with a known concentration (1.00 g/mL) was added to all samples. The concentration of fatty acids in the samples was calculated by normalization with internal standard.

### 2.9. Assessment of Nutritional Quality of Lipids in Fresh Meat

Desirable fatty acids were calculated by summing the content of the following acids: C18:0, monounsaturated, and polyunsaturated fatty acids. The nutritional quality of the lipid fraction of fresh meat was evaluated using the atherogenicity index (AI) and thrombogenicity index (TI), based on the fatty acids found in the samples. The calculations followed the method outlined by Ulbricht and Southgate [27]. After identifying the fatty acids, Δ-9 desaturase indices were determined based on equations from Malau-Aduli et al. [28].

### 2.10. Statistical Analysis

Data on intake, performance, digestibility, and feeding behavior were subjected to analysis of variance (ANOVA) and the F-test with a 5% significance level for type I errors. Variables with significant differences among groups were further analyzed using Tukey’s test with the same level of significance. The analyses were conducted using the PROC GLM procedure in SAS 9.1.3 [29].

## 3. Results

The dry matter allowance ranged from 9.50 to 17.35 kg/BW during the dry and rainy seasons, respectively. Despite this variation, the green leaf availability (kg DM/ha) was similar between the dry (1552 kg DM/ha) and rainy (1874 kg DM/ha) seasons (Table 4).

### 3.1. Intake and Apparent Digestibility

The different post-weaning supplementation strategies did not significantly influence (*p* > 0.05) the intakes of DM (total), forage DM, or organic matter (OM) by the steers. However, steers under the PS1/PS2 post-weaning strategy exhibited higher (*p* < 0.05) crude protein (CP) intakes compared to those under the MS/US and US/PS1 strategies (Table 5).

The supplementation strategies had no significant impact (*p* > 0.05) on the intakes of neutral detergent fiber (NDFap), non-fibrous carbohydrates (NFC), or total digestible nutrients (TDN) by the steers. Likewise, the apparent digestibility of DM, OM, NDF, and NFC did not differ (*p* > 0.05) in response to the supplementation strategies. However, the apparent digestibility of CP from the diet of steers supplemented with PS1/PS2 was higher (*p* < 0.05) compared to those of the other groups.

### 3.2. Feeding Behavior

The grazing time was similar (*p* > 0.05) for steers subjected to the MS/US and US/PS1 strategies but shorter (*p* < 0.05) for those supplemented with PS1/PS2. Steers under the US/PS1 post-weaning strategy spent more time (*p* < 0.05) ruminating and less time idle (Table 6).

Steers subjected to the PS1/PS2 post-weaning strategy spent more time (*p* < 0.05) at the trough and had higher (*p* < 0.05) biting rates compared to steers on US/PS1.

The animals subjected to the PS1/PS2 post-weaning strategy displayed greater feeding and rumination efficiencies (*p* < 0.05) compared with the US/PS1 group.

### 3.3. Performance and Carcass Traits

The supplementation strategies did not affect (*p* > 0.05) the body weight of steers at the end of the post-weaning phase. However, the average daily gain was higher for steers receiving the MS/US post-weaning strategy in comparison to the PS1/PS2 group (Table 7).

During the finishing period, the supplementation strategies had no significant effect (*p* > 0.05) on the slaughter weight. As observed in the post-weaning phase, the average daily gain during the finishing period was also similar between the MS/US and US/PS1 groups, but lower (*p* < 0.05) in those finished with the PS1/PS2 strategy. However, the hot carcass weight and backfat thickness were consistently higher (*p* < 0.05) for steers in the PS1/PS2 post-weaning and finishing strategy compared to the other groups.

### 3.4. Proximate and Fatty Acid Composition of Meat

The post-weaning and finishing strategies did not have a significant effect (*p* > 0.05) on the protein or cholesterol content of the meat from the steers. However, steers subjected to the PS1/PS2 strategy produced meat with a significantly higher (*p* < 0.05) total lipid content (Table 8).

In addition to the total lipid content, the supplementation strategies had a significant influence (*p* < 0.05) on the levels of various fatty acids, including C20:0, C24:0, C14:1, C15:1, C18:1n9t, C18:1n9c, C18:3n3, C18:2c9t11, C18:2t10c12, and C20:5n3, and on the total sum of polyunsaturated fatty acids (Table 9).

The content of desirable fatty acids, as well as the atherogenicity and thrombogenicity indices of the steers’ meat, was not influenced by the post-weaning and finishing supplementation strategies. Furthermore, there was a predominance of ω-6 fatty acids in the meat from steers that received the PS1/PS2 strategy and ω-3 fatty acids in those that received the MS/US strategy (Table 10).

## 4. Discussion

The DM and OM intakes by steers were not significantly influenced by the supplementation strategies. This outcome could be attributed to the consistent availability and quality of the pasture, including green dry matter, green leaves, and pasture protein content, during both the post-weaning and finishing stages [30]. The greater availability of dry matter enabled the steers to select portions of pasture with a higher nutritional value (green leaves), leading to similar forage intakes across the tested strategies [31]. Additionally, the relatively low levels (>1 g/kg BW) of supplementation used (1 and 2 g/kg BW) and the similar composition of the supplements meant that the supplementation had minimal substitutive or additive effects on intake [32]. In a related context, [33] found that, during the rainy season on tropical pasture, steers given either mineral salt with a urea or concentrate supplement at 1 or 2 g/kg BW showed no differences in DM intake, suggesting that forage availability during the rainy season can significantly impact the effects of supplementation in cattle.

Neutral detergent fiber is the major component of forage dry matter, and the intake of this fraction reflected the pattern observed for dietary DM [34]. However, the low levels of NFC in the supplements (SP1 and SP2) could explain why the supplementation strategies had no significant effect on NFC and TDN intakes [35]. In contrast, Rocha et al. [7] reported that using more intensive supplementation strategies increased NFC and TDN intakes among steers finished on tropical pasture throughout the year.

The elevated levels of CP in the supplements (SP1 and SP2), derived from both true protein and non-protein nitrogen (NPN), account for the approximately 100 g/day increase in CP intake among steers following the PS1/PS2 post-weaning and finishing strategy [36]. The CP content in the pasture during both the dry season (95.0 g/kg) and the rainy season (83.0 g/kg) was already sufficient to meet the requirements for adequate ruminal fermentation [37]. However, the provision of true protein (as opposed to solely NPN, available in the urea-based supplement [US]) throughout the dry and rainy seasons (the differential of the PS1/PS2 strategy) could have supported a greater population of bacteria involved in ruminal microbial protein synthesis [38], resulting in higher digestibility, a better amino acid profile, and the increased ruminal production of volatile fatty acids [39].

We anticipated that the US/PS1 and PS1/PS2 strategies would result in greater apparent digestibilities of OM and NDF in the diets. This expectation was based on the well-documented effect of synchrony in ruminal degradation rates between nitrogen sources and organic matter potentially fermentable in cattle rumen [5,40]. However, the high CP content in the pasture, coupled with the provision of forage portions with a superior nutritional value, may have resulted in the apparent digestibilities of OM and NDF in the MS/US strategy being on par with those observed in the US/PS1 and PS1/PS2 strategies, which had a higher CP supply, throughout the post-weaning period [41]. In this respect, the greater apparent digestibility of CP in the diet of steers receiving the PS1/PS2 strategy can be attributed to their higher CP intake and possibly greater synthesis of microbial protein, which has high intestinal digestibility in the rumen of animals under this strategy in the post-weaning phase [42]. Carvalho et al. [43] also reported an increase in CP digestibility when supplementation levels increased from 1 to 2 g/kg BW for calves on tropical pasture during the post-weaning period.

Steers that had protein supplements available throughout the year (PS1/PS2) spent less time grazing and ruminating, possibly because they spent more time at the trough or idle compared to those subjected to the MS/US and US/PS1 strategies [44]. This change in daily grazing patterns for steers in the PS1/PS2 strategy may be related to an increased time spent selecting pasture—an observation supported by their higher biting rate [45]. Furthermore, we propose that the increased consumption of supplement (as inferred from its year-round availability and the prolonged time at the trough), along with the higher level of pasture selection, may have resulted in rumen cuds with a higher ruminal DM content, leading to more chews per cud but lower levels of NDF, thereby reducing chewing time per cud [46]. Rocha et al. [47] also reported a longer chewing time per day and a shorter chewing time per ruminated cud for steers subjected to higher levels of dietary supplementation throughout the year.

Even though the body weight at the end of the post-weaning period and at slaughter was not affected by the supplementation strategies used, the average daily gain (ADG) was lower for steers that underwent post-weaning and finishing with the PS1/PS2 strategy compared to those subjected to MS/US and US/PS1 strategies. Sampaio et al. [9] also observed lower ADG in steers receiving protein–energy supplementation compared to those given only a mineral mixture. The reduced ADG in PS1/PS2 animals may be due to a lower gastrointestinal tract content weight. The ingestion of protein supplements in this strategy could accelerate the passage rate and lead to quicker gastrointestinal content disappearance, resulting in a lower body weight [48]. Moreover, the greater supplement intake throughout the year in the PS1/PS2 strategy might have altered body and gain composition in steers, leading to increased fat deposition in the carcass. This increased energy demand for maintenance and growth adversely affected feed efficiency [49].

On the other hand, the carcass weight and yield of steers finished with PS1/PS2 were consistently higher, likely due to the greater metabolizable protein and energy intake in the PS1/PS2 strategy compared to other strategies [50]. Canozzi et al. [51] also reported that carcass weight increased with higher supplementation levels during the finishing phase. The year-round supplementation in the PS1/PS2 strategy likely resulted in a larger rumen bacterial population, leading to increased microbial protein synthesis and volatile fatty acid production [52]. The latter is associated with lipogenesis processes in bovines, which may explain the higher carcass fatness (thicker subcutaneous fat layer) and greater intramuscular fat in the meat (i.e., more total meat fat) [53]. Additionally, various studies [54,55] indicate that increased concentrate supplementation at pasture finishing correlates with a rise in intramuscular fat in beef.

Steers finished with the MS/US strategy (lower intensification) produced meat with higher levels of C20:0, C24:0, C14:1, C18:1n9c, C18:2c9t11, C18:2t10c12, C18:3, and C20:5n3, and a higher sum of polyunsaturated fatty acids. This increase could be linked to the greater forage intake and absence of concentrate consumption in the MS/US strategy, resulting in a higher intake of C20:0, C24:0, and C18:3—fatty acids commonly found in tropical grasses [56,57]. Furthermore, the higher availability of C18:3 from forage in the rumen of MS/US steers leads to a larger amount of unsaturated fatty acids available for ruminal bacteria to biohydrogenate [58], explaining the elevated concentration of C18:1n9c, C18:2c9t11, and C18:2t10c12, which are conjugated linoleic acid (CLA) isomers [59].

The lower levels of triglycerides in the meat of MS/US steers (see meat fat) could account for the higher levels of C20:5n3, typically associated with phospholipids in myocyte membranes, leading to a relatively higher concentration of phospholipids in the meat of steers with a lower neutral lipid deposition [60]. The greater deposition of C18:3 and C20:5n3 fatty acids explains the higher sum of ω-3 fatty acids and the lower ω-6:ω-3 ratio observed in the meat from MS/US steers. Silva et al. [54] found that meat from steers supplemented only with mineral mixture contained more ω-3 fatty acids and a lower ω-6:ω-3 ratio compared to that of supplemented animals.

On the other hand, the greater ruminal bacterial activity in PS1/PS2 steers may explain the higher presence of odd-chain fatty acids (C15:1), derived from the membrane of the bacteria [61]. Additionally, the greater presence of C18:1n9t in the meat from the steers might be associated with the higher production of trans-11 isomer shifts in the rumen due to the increased starch presence provided by the supplement [62]. The PS1/PS2 strategy also produced meat with a higher ω-6 fatty acid content, likely due to the vegetable oil in the grain-based supplement [63]. Despite the higher ω-6:ω-3 ratio, the PS1/PS2 supplementation strategy produced similar meat in terms of fatty acid profile, atherogenicity, and thrombogenicity indices, demonstrating that increased concentrate supplementation (as used in this study) maintains a desirable fatty acid composition in meat [64].

## 5. Conclusions

Restricted concentrate supplementation (mineral salt and mineral salt with urea weight during the rainy and dry seasons, respectively) in the post-weaning and finishing phases of steers improved the average daily gain and content of polyunsaturated fatty acids and ω-3 fatty acids in the meat. Moderate concentrate supplementation (1 and 2 g/kg of body weight during the rainy and dry seasons, respectively) in the post-weaning and finishing phases of steers improved crude protein intake and digestibility, feeding and rumination efficiency, carcass weight and fatness, and intramuscular fat. However, this strategy reduced the total ω-3 fatty acids and conjugated linoleic acid isomers in the meat. Concentrate supplementation rates of 1 g/kg during the rainy season and 2 g/kg during the dry season are recommended for steers on tropical pasture during the post-weaning and finishing stages.

## Figures and Tables

**Table 1:** Supplementation strategies.

	Restricted (MS/US)	Low (US/PS1)	Moderate (PS1/PS2)
Rainy ^1^	Mineral salt ad libitum	Mineral salt with urea ad libitum	Protein supplement (1 g/kg of BW ^3^)
Dry ^2^	Mineral salt with urea ad libitum	Protein supplement (1 g/kg of BW ^3^)	Protein supplement (2 g/kg of BW ^3^)

^1^ Rainy season, between the months of January and July; ^2^ Dry season, between the months of August and December; ^3^ Body weight.

**Table 2:** Proportion of ingredients in the concentrates (%, dry-matter basis).

	Rainy Season			Dry Season		
	MS ^1^	US ^2^	PS1 ^3^	US	PS1	PS2 ^4^
Grain sorghum	-	-	56.55	-	56.55	49.20
Soybean meal	-	-	19.38	-	19.38	31.34
Urea	-	25	14.93	25	14.93	13.94
Mineral salt ^5^	100	75	9.14	75	9.14	5.54

^1^ Mineral salt ad libitum; ^2^ Mineral salt with urea ad libitum; ^3^ Protein supplement (1 g/kg of body weight); ^4^ Protein supplement (2 g/kg of body weight). ^5^ Composition: calcium 235 g; phosphorus 60 g; magnesium 16 g; sulfur 12 g; cobalt 150 mg; copper 1600 mg; iodine 190 mg; manganese 1400 mg; iron 1000 mg; selenium 32 mg; zinc 6000 mg; 1120 mg; fluoride (maximum) 1600 mg.

**Table 3:** Chemical composition (g/kg) of forage (*Urochloa brizantha* cv. Marandu) and concentrate supplements used in the experiment.

	Forage (Simulated Grazing)		Protein Supplement Level	
	Rainy	Dry	1 g/kg	2 g/kg
Dry matter	263.2	400.0	895.9	897.7
Organic matter	912.9	889.0	894.4	886.6
Ash	87.2	110.0	105.5	113.4
Crude protein	95.0	83.0	465.8	397.6
Ether extract	16.6	22.6	32.9	30.9
Total carbohydrates	805.9	778.0	397.9	458.1
NDFap	696.0	653.5	254.6	336.6
NFC	156.9	129.5	160.7	121.5
iNDF	185.0	250.0	12.2	13.8
TDN	509.6	489.8	613.8	653.1

NFC: non-fibrous carbohydrates; NDFap: neutral detergent fiber corrected for ash and protein; iNDF: indigestible neutral detergent fiber; TDN: total digestible nutrients.

**Table 4:** Canopy characteristics of *Urochloa brizantha* cv. Marandu pasture.

Characteristics of the Forage Canopy	Season	
	Dry	Rainy
Total dry matter (kg/ha)	4236	6319
Potentially digestible dry matter (kg DM ^1^/ha)	3446	5205
Green dry matter (kg/ha)	3098	3973
Stem + sheath (kg DM/ha)	1538	2099
Green leaf (kg DM/ha)	1552	1874
Senescent material (kg DM/ha)	1147	1296
Dry matter allowance (kg/BW ^2^)	9.50	17.35

^1^ Dry matter; ^2^ Body weight.

**Table 5:** Intake and apparent digestibility of nutrients from the diet of steers * receiving supplement-reduction strategies on tropical pasture during the post-weaning period.

	Supplementation Strategy			SEM ^4^	*p*-Value ^5^
	MS/US ^1^	US/PS1 ^2^	PS1/PS2 ^3^		
Intake (kg/day)					
Total dry matter	7.41	7.42	7.79	1.016	0.613
Forage dry matter	7.41	7.42	7.42	0.991	0.999
Organic matter	6.83	6.83	7.16	0.935	0.631
Crude protein	0.74 ^b^	0.77 ^b^	0.91 ^a^	0.122	0.002
Ether extract	0.13	0.13	0.14	0.018	0.205
NDFap ^6^	5.01	5.01	5.12	0.676	0.911
NFC ^7^	1.15	1.15	1.21	0.157	0.615
TDN ^8^	3.87	3.95	4.23	0.667	0.435
Digestibility (%)					
Dry matter	48.71	49.72	51.20	3.357	0.230
Organic matter	51.50	52.84	54.11	3.248	0.185
Crude protein	41.03 ^b^	41.60 ^b^	48.77 ^a^	3.806	0.000
Ether extract	67.03	69.85	72.88	5.671	0.069
NDFap ^6^	54.02	55.51	55.61	3.424	0.482
NFC ^7^	57.14	56.10	56.52	4.227	0.846
TDN ^8^	52.22	53.23	54.30	3.177	0.275

* The animals weighed on average, 378.3 kg at the time of intake. ^1^ Mineral salt ad libitum in the rainy season/Mineral salt with urea in the dry season; ^2^ Mineral salt with urea in the rainy season/Protein supplement (1 g/kg of body weight) in the dry season; ^3^ Protein supplement (1 g/kg body weight) in the rainy season/Protein supplement (2 g/kg body weight) in the dry season. ^4^ Standard deviation of the mean; ^5^ Means followed by letters differ statistically at a 5% probability level using Tukey’s test; ^6^ Neutral detergent fiber corrected for ash and protein; ^7^ Non-fiber carbohydrates; ^8^ Total digestible nutrients.

**Table 6:** Feeding behavior of steers * receiving supplement-reduction strategies on tropical pasture during the post-weaning period.

	Supplementation Strategy			SEM ^4^	*p*-Value ^5^
	MS/US ^1^	US/PS1 ^2^	PS1/PS2 ^3^		
Grazing time (min)	497.00 ^a^	534.00 ^a^	437.00 ^b^	76.923	<0.0001
Rumination time (min)	365.00 ^b^	410.00 ^a^	352.00 ^b^	65.366	0.0013
Idle time (min)	572.00 ^b^	488.00 ^c^	640.00 ^a^	94.350	<0.0001
Time at trough (min)	4.50 ^b^	7.10 ^b^	10.60 ^a^	5.671	<0.0002
Biting time (s)	22.00	23.00	22.00	2.667	0.4701
Biting rate (n/min)	49.00 ^ab^	470.00 ^b^	52.00 ^a^	8.337	0.0311
Ruminated cuds (n/day)	395.00 ^b^	463.00 ^a^	424.00 ^ab^	105.85	0.0378
Chewing (n/cud)	50.00 ^b^	53.00 ^ab^	58.00 ^a^	10.953	0.0262
Chewing (s/cud)	57.00 ^a^	55.00 ^ab^	51.00 ^b^	8.552	0.0147
Feeding efficiency (kg DM ^6^/h)	0.92 ^ab^	0.84 ^b^	1.07 ^a^	0.245	0.0009
Feeding efficiency (kg NDFap ^7^/h)	0.62 ^ab^	0.57 ^b^	0.70 ^a^	0.163	0.0046
Rumination efficiency (kg DM/h)	1.23 ^ab^	1.12 ^b^	1.37 ^a^	0.260	0.0010
Rumination efficiency (kg NDFap/h)	0.83 ^ab^	0.76 ^b^	0.90 ^a^	0.172	0.0057

* The animals weighed on average, 378.3 kg at the time of intake. ^1^ Mineral salt ad libitum in the rainy season/Mineral salt with urea in the dry season; ^2^ Mineral salt with urea in the rainy season/Protein supplement (1 g/kg of body weight) in the dry season; ^3^ Protein supplement (1 g/kg body weight) in the rainy season/Protein supplement (2 g/kg body weight) in the dry season. ^4^ Standard deviation of the mean; ^5^ Means followed by letters differ statistically at a 5% probability level using Tukey’s test; ^6^ Dry matter; ^7^ Neutral detergent fiber corrected for ash and protein.

**Table 7:** Performance and carcass traits of steers receiving supplement-reduction strategies on tropical pasture during the post-weaning and finishing periods.

	Supplementation Strategy			SEM ^4^	*p*-Value ^5^
	MS/US ^1^	US/PS1 ^2^	PS1/PS2 ^3^		
Post-weaning (240 days)					
Initial body weight (kg)	332.41	331.91	340.82	44.762	0.8770
Final body weight (kg)	424.86	412.68	415.45	50.788	0.8410
Average daily gain (kg/day)	0.880 ^a^	0.769 ^ab^	0.710 ^b^	0.148	0.0350
Feed efficiency	0.120 ^a^	0.100 ^ab^	0.090 ^b^	0.019	0.0060
Finishing (70 days)					
Initial body weight (kg)	430.64	426.45	455.27	52.057	0.3857
Final body weight (kg)	477.30	491.20	502.70	50.805	0.5506
Average daily gain (kg/day)	0.61 ^a^	0.69 ^a^	0.45 ^b^	0.181	0.0043
Hot carcass weight (kg)	232.83 ^c^	253.27 ^b^	267.44 ^a^	12.082	<0.0001
Hot carcass yield (%)	50.23 ^b^	50.65 ^b^	52.48 ^a^	2.014	<0.0001
Ribeye area (cm^2^)	65.64 ^b^	73.25 ^a^	74.37 ^a^	3.689	<0.0001
Backfat thickness (mm)	3.32 ^c^	3.78 ^b^	4.06 ^a^	0.224	<0.0001

^1^ Mineral salt ad libitum in the rainy season/Mineral salt with urea in the dry season; ^2^ Mineral salt with urea in the rainy season/Protein supplement (1 g/kg of body weight) in the dry season; ^3^ Protein supplement (1 g/kg body weight) in the rainy season/Protein supplement (2 g/kg body weight) in the dry season. ^4^ Standard deviation of the mean; ^5^ Means followed by letters differ statistically at a 5% probability level using Tukey’s test.

**Table 8:** Proximate composition and cholesterol content of meat (*Longissimus dorsi*) from steers finished with supplement-reduction strategies on tropical pasture.

	Supplementation Strategy			SEM ^4^	*p*-Value ^5^
	MS/US ^1^	US/PS1 ^2^	PS1/PS2 ^3^		
Moisture (%)	71.03	70.57	70.50	1.878	0.3507
Ash (%)	3.12	3.48	3.56	0.182	0.1108
Protein (%)	23.55	22.99	22.03	2.057	0.0683
Total lipids (%)	2.25 ^b^	3.00 ^b^	3.93 ^a^	0.607	0.0001
Cholesterol (mg/100 g)	43.48	43.03	44.08	3.561	0.1453

^1^ Mineral salt ad libitum in the rainy season/Mineral salt with urea in the dry season; ^2^ Mineral salt with urea in the rainy season/Protein supplement (1 g/kg of body weight) in the dry season; ^3^ Protein supplement (1 g/kg body weight) in the rainy season/Protein supplement (2 g/kg body weight) in the dry season. ^4^ Standard deviation of the mean; ^5^ Means followed by letters differ statistically at a 5% probability level using Tukey’s test.

**Table 9:** Fatty acid composition (g/100 g) of meat (*Longissimus dorsi*) from steers finished with supplement-reduction strategies on tropical pasture.

	Supplementation Strategy			SEM ^4^	*p*-Value ^5^
	MS/US ^1^	US/PS1 ^2^	PS1/PS2 ^3^		
Saturated fatty acids (SFA)					
C6:0	0.02	0.02	0.03	0.006	0.6410
C8:0	0.01	0.01	0.01	0.003	0.3349
C10:0	0.06	0.05	0.06	0.012	0.8712
C12:0	0.07	0.07	0.08	0.014	0.5080
C14:0	2.69	2.70	2.37	0.457	0.4544
C15:0	0.54	0.59	0.57	0.068	0.0608
C16:0	23.60	24.53	24.30	1.753	0.3058
C17:0	1.31	1.32	1.31	0.090	0.0660
C18:0	23.66	24.76	24.66	6.757	0.0585
C20:0	1.98 ^a^	1.35 ^b^	1.43 ^b^	0.325	0.0359
C21:0	0.05	0.06	0.06	0.010	0.0878
C22:0	0.14	0.14	0.13	0.039	0.8960
C24:0	0.04 ^a^	0.02 ^b^	0.02 ^b^	0.013	0.0176
∑SFA	54.17	55.62	55.03	2.610	0.2585
Monounsaturated fatty acids (MUFA)					
C14:1	0.46 ^a^	0.33 ^b^	0.28^b^	0.073	0.0217
C15:1	0.26 ^b^	0.32 ^a^	0.34^a^	0.037	0.0146
C16:1	2.94	2.95	3.10	0.556	0.0888
C17:1	0.65	0.65	0.64	0.054	0.9214
C18:1n9t	2.58 ^b^	2.93 ^ab^	3.36 ^a^	0.385	0.0229
C18:1n9c	34.91 ^a^	33.48 ^b^	33.39 ^b^	2.534	0.0382
C20:1	0.18	0.19	0.18	0.034	0.8872
C22:1n9	0.04	0.03	0.04	0.013	0.3904
C24:1	0.23	0.24	0.30	0.061	0.9520
∑MUFA	42.25	41.12	41.63	2.563	0.3749
Polyunsaturated fatty acids (PUFA)					
C18:2n6t	0.14	0.14	0.15	0.015	0.1079
C18:2n6c	0.35	0.35	0.35	0.058	0.9775
C18:3n6	0.18	0.17	0.17	0.024	0.4109
C18:3n3	0.70 ^a^	0.53 ^b^	0.52 ^b^	0.075	0.0039
C18:2c9t11	0.51 ^a^	0.42 ^b^	0.38 ^b^	0.045	0.0459
C18:2t10c12	0.12 ^a^	0.10 ^b^	0.10 ^b^	0.013	0.0370
C20:2	0.05	0.04	0.04	0.010	0.0659
C20:3n6	0.11	0.11	0.10	0.022	0.6129
C20:4n6	0.82 ^b^	0.93 ^b^	1.09 ^a^	0.182	0.0040
C22:2	0.28	0.28	0.27	0.095	0.9171
C20:5n3	0.28 ^a^	0.15 ^b^	0.14 ^b^	0.058	0.0040
C22:6n3	0.04	0.04	0.03	0.010	0.0467
∑PUFA	3.58 ^a^	3.26 ^b^	3.34 ^b^	0.218	0.0153

^1^ Mineral salt ad libitum in the rainy season/Mineral salt with urea in the dry season; ^2^ Mineral salt with urea in the rainy season/Protein supplement (1 g/kg of body weight) in the dry season; ^3^ Protein supplement (1 g/kg body weight) in the rainy season/Protein supplement (2 g/kg body weight) in the dry season. ^4^ Standard deviation of the mean; ^5^ Means followed by letters differ statistically at a 5% probability level using Tukey’s test.

**Table 10:** Nutritional quality indices and desaturase activity indices of meat (*Longissimus dorsi*) from steers finished with supplement-reduction strategies on tropical pasture.

	Supplementation Strategy			SEM ^4^	*p*-Value ^5^
	MS/US ^1^	US/PS1 ^2^	PS1/PS2 ^3^		
MUFA:SFA ^6^	0.78	0.74	0.76	0.090	0.3455
PUFA:SFA ^7^	0.07	0.06	0.06	0.005	0.1745
DFA ^8^	69.49	69.14	69.63	2.145	0.6815
Atherogenicity index	0.77	0.81	0.77	0.101	0.5509
Thrombogenicity index	1.88	2.00	1.94	0.165	0.0949
h:H ^9^	1.51	1.41	1.46	0.187	0.5035
∑ ω-6 (n-6) fatty acids	1.60 ^b^	1.70 ^b^	1.86 ^a^	0.214	0.0118
∑ ω-3 (n-3) fatty acids	1.02 ^a^	0.72 ^b^	0.69 ^b^	0.100	0.0003
n-6:n-3	1.57 ^b^	2.36 ^a^	2.69 ^a^	0.465	0.0031
Δ^9^ desaturase 14	14.60 ^a^	10.89 ^b^	10.56 ^b^	1.563	0.0100
Δ^9^ desaturase 16	11.07	10.73	11.35	1.041	0.3488
Δ^9^ desaturase 18	59.60 ^a^	57.48 ^b^	57.52 ^b^	2.974	0.0349

^1^ Mineral salt ad libitum in the rainy season/Mineral salt with urea in the dry season; ^2^ Mineral salt with urea in the rainy season/Protein supplement (1 g/kg of body weight) in the dry season; ^3^ Protein supplement (1 g/kg body weight) in the rainy season/Protein supplement (2 g/kg body weight) in the dry season. ^4^ Standard deviation of the mean; ^5^ Means followed by letters differ statistically at a 5% probability level using Tukey’s test. ^6^ Ratio of monounsaturated to saturated fatty acids; ^7^ Ratio of polyunsaturated to saturated fatty acids; ^8^ Desirable fatty acids; ^9^ Ratio of hypocholesterolemic to hypercholesterolemic fatty acids.

## Data Availability

None of the data were deposited in an official repository.
